# Health literacy and health outcomes in patients with low back pain: a scoping review

**DOI:** 10.1186/s12911-021-01572-0

**Published:** 2021-07-13

**Authors:** Ye King Clarence See, Helen Elizabeth Smith, Lorainne Tudor Car, Joanne Protheroe, Wei Cong Wong, Bernadette Bartlam

**Affiliations:** 1 Lee Kong Chian School of Medicine, Nanyang Technological University, Singapore, Singapore; 2 School of Primary, Community and Social Care, Keele University, Newcastle, UK

**Keywords:** Core Outcome Set, Health outcomes, Chronic pain, Disability, Musculoskeletal conditions

## Abstract

**Background:**

Low back pain is a leading cause of disability worldwide. Health literacy has been associated with pain intensity and pain control. However, there is a paucity of evidence regarding this association. In the field of low back pain research, inconsistent reporting of outcomes has been highlighted. To address this issue a Core Outcome Set has been developed.

**Objectives:**

The objectives of this scoping review were: (1) The health literacy measures currently employed for low back pain and the aspects of health literacy they include. (2) The low back pain health outcomes included in such work. (3) The extent to which these health outcomes reflect the Core Outcome Set for Clinical Trials in Non-Specific Low Back Pain.

**Methods:**

The search included thirteen bibliographic databases, using medical subject heading terms for low back pain and health literacy, and followed the Preferred Reporting Items for Systematic Reviews and Meta-Analyses extension for Scoping Reviews guidelines. The eligibility criteria were defined by the Joanna Briggs Institute PCC mnemonic. A thematic framework approach was used for analysis.

**Results:**

The search yielded ten relevant studies for inclusion, amongst which a total of nine health literacy measures and 50 health outcome measures were used. Most health literacy measures focused on functional health literacy, with few assessing communicative and critical health literacy. The health outcomes assessed by the included studies could be broadly categorised into: Pain, Disability, Behaviour, Knowledge and Beliefs, and Resource Utilisation. Most of these outcome measures studied (36 out of 50) did not directly reflect the Core Outcome Set for Clinical Trials in Non-Specific Low Back Pain.

**Conclusions:**

To allow for comparison across findings and the development of a rigorous evidence base, future work should include the Core Outcome Set for Clinical Trials in Non-Specific Low Back Pain. There is an urgent need to broaden the evidence-base to include regions where low back pain morbidity is high, but data is lacking. Such work demands the incorporation of comprehensive measures of health literacy that have both generic and culturally sensitive components.

## Background

Low back pain (LBP) is the single leading cause of disability globally and is rising [1, 2]. In 2017, the point prevalence of LBP was estimated to be 7.5% the global population, or approximately 577 million people [3]. Financial costs from LBP are estimated to be in the order of billions of US dollars (USD) [2, 4], while the economic burden of members of the workforce suffering from LBP is estimated in the USA alone to be USD 7.4 billion/year [5]. Traditionally conceptualised as solely secondary to mechanical injury, LBP is now described within a bio-psychosocial model, resulting from an interaction of physical, psychological and social influences [6]. Risk factors for LBP include an older age, increased psychological or psychosocial stress, a lower socioeconomic status, and a lower educational status [7, 8].

Effective self-management is crucial to improving LBP outcomes [9, 10]. Studies have also demonstrated the need to focus on health literacy (HL) in order to develop effective patient education materials and/or patient resources to support self-management in such patients [11, 12]. The concept of HL is extensive and incorporates functional, communicative and critical domains [13]. It is defined as “the achievement of a level of knowledge, personal skills and confidence to take action to improve personal and community health by changing personal lifestyles and living conditions” [14]. At its core is an observable set of skills that can be developed and improved through effective communication and education to enhance autonomy and empower people to make decisions relating to their health and changing circumstances [14, 15]. At the inaugural Outcome Measures in Rheumatology Clinical Trials (OMERACT) Health Literacy Special Interest Group workshop, 16 themes at the micro, meso and macro level were identified, including cognitive capacity, access to information, and health systems [16]. Independent of other socio-demographic factors, low HL is associated with higher mortality amongst older people, poorer health outcomes, and higher morbidity [17–19]. On the other hand, higher HL is linked to lower pain intensity and better pain control among those with chronic pain [1, 20].

Despite the need for a stronger evidence base in LBP management, inconsistent reporting of outcomes in clinical trials of patients with LBP has been highlighted [21]. This potentially hinders the comparison of findings across studies and the reliability of systematic reviews. To address this issue a core outcome set (COS) has been developed, led by an International Steering Committee, defining the minimum set of outcomes that should be reported in all clinical trials. The COS includes 'physical functioning', 'pain intensity', 'health-related quality of life' and 'number of deaths' [22].

There also exists a paucity of research to underpin evidence-based practice of LBP treatment in low- and middle-income countries (LMIC) [23, 24]. This is a substantial knowledge gap given the significance of LBP in LMICs. Asia alone has the largest number of LBP disability-adjusted life years internationally and the highest risk of occupational LBP is in the agricultural sector—a major sector in Asian economies [25]. Existing evidence tends to be from high income countries and cannot be accurately applied to the LMIC context, given that pain reporting, manifestation and management is influenced by socio-cultural and genetic factors [26].

To develop more evidence-based interventions and guidelines we need to better understand the relationship between HL and LBP outcomes. An initial scoping search of the literature was conducted to assess whether reviews and guidelines on this topic have already been published and what was lacking. This only yielded a single systematic review by Edward et al. in 2018 on the impact of HL on LBP management. The study identified only three relevant studies, all of which were based in high income Western nations. However, the authors of the review acknowledged “possible incomplete retrieval of identified research and reporting bias” [27] as the search was limited to four bibliographic databases and limits were also placed on year of publication, language, and article formats, amongst other search filters [27].

This scoping review builds on Edward et al.’s work and had three objectives. These were to methodically map evidence on:

1. The health literacy measures currently employed for low back pain and the aspects of health literacy they include.
2. The low back pain health outcomes included in such work.
3. The extent to which these health outcomes reflect the Core Outcome Set for Clinical Trials in Non-Specific Low Back Pain.

Scoping reviews are used instead of systematic reviews where the purpose of the review is to identify knowledge gaps, scope a body of literature, clarify concepts or to investigate research conduct [28]. This methodology was chosen in the light of the paucity of existing literature and to reflect and build from the limitations encountered in the work of Edward et al. [27]. To do so, this scoping study expanded the search from four to 13 bibliographic databases and did not utilise search limiters or filters such as time or language filters. Unlike the systematic review carried out by Edward et al. [27], this study is a scoping review with the emphasis on identifying the variety of HL and LBP outcome measures employed in existing literature, rather than reporting the degree of association between HL and LBP health outcomes. The aim in doing so is to provide a critique on the choice of outcomes studied and measures used, and to identify implications for future research.

## Materials and methods

### Literature search strategy

The searches were conducted in: MEDLINE, Pubmed, Academic Search Complete, The Cumulative Index to Nursing and Allied Health Literature, Education Source, Education Resource Information Centre, PsycINFO, Global Health, Embase (Ovid)**,** Web of Science, Cochrane, Google Scholar, and ClinicalKey.

MeSH (medical subject heading) terms used included: *Back Pain, Back Ache, Back Pain with Radiation, Back Pain without Radiation, Backache, Vertebrogenic Pain Syndrome, Low Back Pain, Low Back Ache, Low Back Pain Mechanical, Low Back Pain Posterior Compartment, Low Back Pain Postural, Low Back Pain Recurrent, Low Backache, Lower Back Pain, Lumbago, Mechanical Low Back Pain, Postural Low Back Pain, Recurrent Low Back Pain*.

The MeSH term used to search for HL was *Health Literacy*. No additional search filters were applied. See “Appendix 1” for an example of a search strategy. The search was conducted in August 2019. It was updated in February 2021, reflecting the peer-review process in the context of COVID-19, and no additional studies were identified as meeting the inclusion criteria.

The search strategy was developed in consultation with the library team at the University, as well as expert opinion within the research team, which consisted of a range of expert researchers and clinicians [29]. This included BB (social sciences, primary care research, musculoskeletal research, patient perspectives and health literacy), HES (primary care clinician, health services research, evidence-based medicine), LTC (primary care clinician, health services research, evidence-based medicine, and systematic reviews) and JP (primary care clinician, musculoskeletal conditions, health services research and health literacy).

### Inclusion and exclusion criteria

The Joanna Briggs Institute (JBI) manual’s PCC mnemonic [30] was used to clarify the research focus in formulating the inclusion and exclusion criteria (Table 1):

**Table 1:** Inclusion and exclusion criteria

Inclusion criteria	Exclusion criteria
Patients with LBP (≥ 10% of study population), of any age, gender, or race	Non-research or Non-peer reviewed sources of evidence (e.g. grey literature, policy documents, expert opinions, guidelines)
Any healthcare setting, in any geographical setting	Studies only analysing generic literacy, numeracy, and education level not in the context of healthcare
Any peer reviewed research study (of any study design)	
Utilisation of specific HL and LBP health outcome measures

- Population—Patients with LBP (≥ 10% of study population), of any age, gender, or race
- Concept—Relationship of LBP health outcomes to HL
- Context—Any healthcare setting, in any geographical setting

Only research studies were included in this scoping review as the objectives of this study focused on measures used in LBP research. Hence other sources of evidence (e.g. grey literature, policy documents, expert opinions, guidelines) were not included. In addition, studies for inclusion required the use of specific HL and health outcome measures. Studies were excluded if they only analysed generic literacy, numeracy, and education level not in the context of healthcare. Generic patient education interventions have the potential to influence non-HL related determinants of LBP, hence drawing conclusions about HL’s effects on LBP from these studies may be inaccurate [31], and for this reason these studies were excluded.

### Study selection, data extraction and analysis

The search strategy followed the Preferred Reporting Items for Systematic Reviews and Meta-Analyses extension for Scoping Reviews (PRISMA-ScR) guidelines [32]. An independent review of titles and abstracts from the initial search was conducted by two reviewers (CS and WWC). Any discrepancies were resolved through discussion between reviewers, with a third reviewer (HES) included when necessary. However, no discrepancies which could not be resolved between reviewers were encountered. Studies then underwent a full-text review if they investigated a relationship between HL and LBP outcomes.

Data extraction included determinants of HL (age, gender, race, and education level) [33], study design, and types of measures used. Thematic analysis as advocated by Levac et al. [34] was done by adopting a framework analysis approach [35, 36]. The health outcomes used were collated and coded into descriptive themes, and then grouped into overarching categories. These categories were then mapped against the Core Outcome Set for Clinical Trials in Non-Specific Low Back Pain [22], namely 'physical functioning', 'pain intensity', 'health-related quality of life' and 'number of deaths'. The HL measures used were categorised according to their validated component of HL, or if absent, the intention of that specific study. This was done using the classification proposed by Nutbeam, namely functional, communicative and critical HL [37, 38]. The components of this classification have a graded order of complexity, functional HL being the most basic, and critical HL being the most advanced [37]. Functional HL encompasses basic skills in reading and writing, which are important for instance in understanding prescriptions or medicine labels. Communicative HL includes social skills and advanced cognitive and literacy skills to actively participate in daily activities. It is important for example in building up rapport with a social support group. It is also crucial in the doctor-patient relationship, as evidenced by HL tools such as Teach Back aiming to facilitate this [39]. Critical HL comprises the use of even more advanced cognitive and social skills to exert great control over life events and situations. An example of operationalising critical HL is organising social advocacy health promotion within communities, to enable and empower individuals to ‘judge, sift and use’ health information in the context of their own lives and social worlds [40].

As this was a scoping review, grading of evidence was not conducted. Instead, this study followed the Preferred Reporting Items for Systematic Reviews and Meta-Analyses extension for Scoping Reviews (PRISMA-ScR) guidelines [32], as detailed in “Appendix 2”.

## Results

### Articles reviewed

**Fig. 1:**
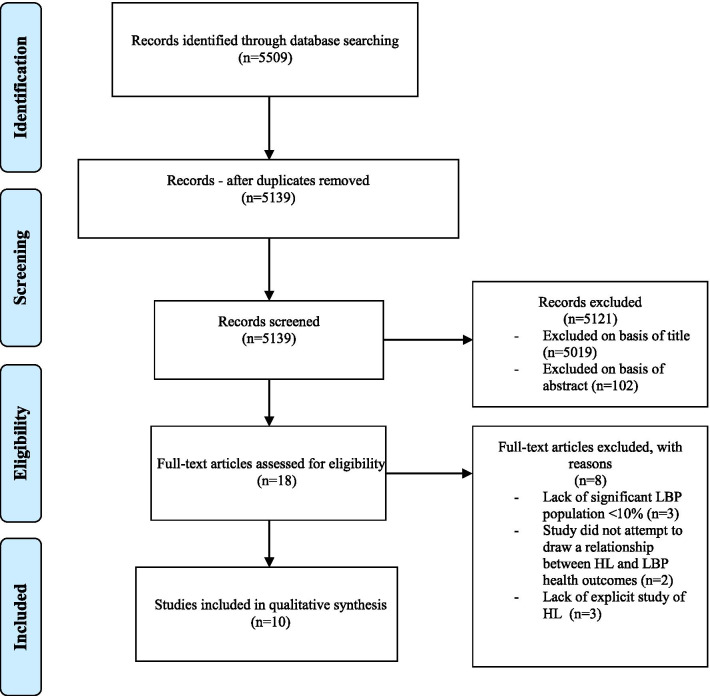
Preferred Reporting Items for Systematic Reviews and Meta-Analyses flow diagram of the literature review

The initial search yielded 5509 ‬articles. After removing duplicates and reviewing titles and abstracts, 18 articles remained for full-text review. Ten of these were included in the final analysis (Fig. 1). The key excluded sources with rationale for their exclusion are listed in “Appendix 3”. Both 2010 and 2011 papers by Briggs et al. [10, 41] were included and recorded as separate studies, as each publication studied different HL measures.

### Summary of key data retrieved from full text reviews

Despite no restrictions being placed on the year of publication, all studies meeting the inclusion criteria were published in 2010 or later, and were all cross-sectional in design (Table 2). They utilised structured questionnaires, apart from one mixed methods study which also used interviews. In terms of country of origin, two studies were conducted in Australia, four in the United States of America, three European studies across four centres (Germany, Austria, Switzerland and Italy), one in Saudi Arabia. Five of the cross-sectional studies solely studied patients with LBP, while the other five studies included LBP as a significant portion of their patient population (> 10%), one of which provided a subpopulation analysis of patients with LBP.

**Table 2:** Summary of study characteristics and population

References	Country	Study design	Study size	Age	Gender	Race	Education level
Briggs et al. [10] Briggs et al. [41]	Australia	Cross-sectional, mixed methods study	n = 117	Mean ages: 38.5 (No CL back pain), 37.4 (CL back pain-low disability), 43.2 (CL back pain-high disability)	Female (n = 71)	Unspecified	≤ Secondary school (n = 17), Trade certificate or diploma (n = 33), University degree (n = 30), No response (n = 37)
Devraj et al. [12]	USA	Cross-sectional	n = 139	≥ 18 years	Females (n = 105)	White (n = 98), African American (n = 24), Hispanic (n = 8), Asian or Pacific Islander (n = 4), Native American (n = 4)	≤ Secondary school (n = 37), Some college (n = 48), University degree (n = 54)
Farin et al. [11]	Germany	Cross-sectional	n = 577	17–85 years	Females (n ~ 317)	Unspecified	≥ Secondary school (77.9%), University-entrance diploma or technical college qualification (20.9%)
Camerini and Schulz [51]	Switzerland and Italy	Cross-sectional	n = 273	20–89 years	Female (n = 159)	Unspecified	≤ Secondary school (n = 90), Post-secondary non tertiary educational degree (n = 145), University degree (n = 38)
Burke et al. [53]	USA	Cross-sectional, retrospective	n = 23,393 (back pain sub-sample of 2580)	≥ 18 years	Unspecified	Included: White, Black, and Others	Included: ≤ High school, ≥ Some college
Al-Eisa et al. [48]	Saudi Arabia	Cross-sectional	n = 227	20–55 years	Female only	Unspecified	Unspecified
MacLeod et al. [54]	USA	Cross-sectional, retrospective	n = 7334	≥ 65 years	Females (n = 4384)	Sicker population (minority/non-white 7.3%, White 92.7%). Healthier population (minority/non-white 3.4%, White 96.6%)	Sicker population (≤ High school 41.8%, ≤ 2 year college 29.0%, ≥ 4 year college 29.2%). Healthier population (≤ High school 39.2%, ≤ 2 year college 28.9%, ≥ 4 year college 32.0%)
Köppen et al. [20]	Austria	Cross-sectional	n = 121	18–65 years	Female (n = 89)	Unspecified	Compulsory school (17%), School leaving examination/ apprenticeship (61%), University (22%)
Glassman et al. [47]	USA	Cross-sectional	n = 186	≥ 18 years	Females (n = 119)	Unspecified	≤ Secondary school (n = 108), University degree (n = 51), no data (n = 27)

All ten studies focused on adult populations (≥ 18 years) and had a majority female population, with one study having entirely female participants. Racial break-down was only provided by two USA studies, both of which had predominantly white study populations. Nine studies collected data on education level, most reporting an even spread across participants.

### Summary of HL measures used

Nine different HL measures were used across the ten studies (Table 3). Most HL measures assessed functional HL, while the number that evaluated communicative and critical HL were fewer than half (Table 4).

**Table 3:** HL and LBP health outcome measures used and their relationship

HL measure	Health outcome (HO)	HO measure	Relationship between HO and HL
Briggs et al. [10]			
Short-form Test of Functional Health Literacy in Adults (S-TOFHLA)	Pain severity	Numeric pain-rating scale	Unspecified
	Pain impact	LBP episodes (last 1 year), workdays missed, sought health professional advice, medication use, intrusion on regular daily and recreational activities	Unspecified
	LBP related disability	Oswestry Disability Index (ODI)	Unspecified
	Fear avoidance	Fear Avoidance Beliefs Questionnaire (FABQ)	No significant relationship
	Beliefs about LBP	Back Pain Beliefs Questionnaire (BBQ)	No significant relationship
	Catastrophising	Coping Skills Questionnaire (CSQ)	No significant relationship
	Beliefs on “cause and course of low back pain”, and “seeking, understanding and utilising low back pain information”	Telephone interviews	Unspecified correlation to HL. However, participants reported obstacles in seeking, comprehending and using LBP information, which were not reflected in S-TOFHLA scores
Briggs et al. [41]			
Health Literacy Measurement Scale (HeLMS)	Same as Briggs 2010	Same as Briggs 2010	Chronic LBP associated with lower scores in HeLMS domain 1: ‘Patient attitudes towards their health’ and greater difficulty in managing personal health
Devraj et al. [12]			
Newest Vital Sign (NVS)	Pain awareness and medication knowledge	12-question survey based on chronic pain guidelines, patient education resources, and previous studies	Limited HL associated with lower ability to find healthcare providers to manage chronic pain, less likely to know alternative methods to treat pain besides medications alone, and less likely to know over-the-counter medications to take for pain control
	Pain severity	100 mm Visual Analogue Scale (VAS)—(pain severity over the past week)	No significant relationship
Farin et al. [11]			
HELP questionnaire (health education literacy of patients with chronic musculoskeletal diseases)	General health status	One-item measure (How would you rate your health?)	Poor self-rated health status was the greatest risk factor for low HL. Study considered this is a causal path in the opposite direction: low HL patients are at a disadvantage and thus experience a less positive disease course
Camerini and Schulz [51]			
Low Back Pain Knowledge Questionnaire	Patient empowerment	Psychological Empowerment Scale	No significant relationship
	Patient involvement	Modified Patients’ Perceived Involvement in Care Scale (M-PICS)	Low HL group more inclined to ask healthcare provider for information regarding treatment plan
	Medication non-adherence	Pain Medication Questionnaire	No significant relationship
	Health outcomes	6 questions from the Chronic Pain Grading Scale on intensity and functionality	No significant relationship
Burke et al. [53]			
“Never heard of it/Do not know much about it” questions from the NHIS (National Health Interview Survey)	Lack of need	“Do not need it” from the NHIS (National Health Interview Survey)	No significant relationship
	Health status	Self-reported health status, functional limitation, hospitalization and emergency department attendance (last 12 months)	No significant relationship
	Health behaviours	Activity level, smoking status, alcohol consumption level, body mass index, flu immunisation (last 12 months), use of pneumonia vaccine	Low HL associated lower activity level
	Healthcare access	Healthcare provider visits (last 12 months), health insurance coverage, delayed healthcare due to cost concerns, delayed healthcare due to non-cost concerns, ability to afford common supplementary healthcare	Low HL associated with greater inability to afford ancillary care
Al-Eisa et al. [48]			
Newest Vital Sign (NVS)	Disability level for LBP	Oswestry Disability Index	Disability was negatively correlated with HL
	Avoidance behaviour due to pain	Fear Avoidance Beliefs' Questionnaire (FABQ)	Negative correlation between FAB (in terms of Physical Activity) and HL No significant relationship between FAB (in terms of work) and HL
MacLeod et al. [54]			
“How confident are you filling out medical forms by yourself?” screening question	Patient dissatisfaction	Modified Consumer Assessment of Healthcare Providers and Systems (CAHPS) survey. 10-point scales measuring dissatisfaction with general healthcare, specialists, physicians, and AARP Medicare Supplement Insurance plans	Inadequate HL associated with greater dissatisfaction with healthcare system and general healthcare (e.g. physicians, specialists, insurers, and general experiences)
	Preventive services or quality of care	Administrative medical claims databases	Inadequate HL associated with reduced compliance towards preventive healthcare services and less uptake of flu immunisations
	Healthcare utilization and expenditures	Administrative medical claims databases	Inadequate HL associated with higher emergency department attendance, inpatient admission and yearly healthcare expenditure
Köppen et al. [20]			
3 screening questions from *Brief Questions to Identify Patients with Inadequate Health Literacy*: “how often do you have someone help you read hospital materials”, “how confident are you filling out medical forms by yourself” and “how often do you have problems learning about your medical condition because of difficulty understanding written information?”	Pain intensity	Visual Analogue Scale (VAS)	Higher HL associated with lower pain intensity
	Pain perception	Short-form McGill Pain Questionnaire (SF-MPQ)	No significant relationship
	Pain duration	Listed in months	No significant relationship
Glassman et al. [47]			
Newest Vital Sign (NVS)	LBP related disability	Oswestry Disability Index	No significant relationship
Health Literacy Assessment (HLA)	Pain	Numeric Rating Scales for Back and Leg Pain	Lower HL associated with higher back pain scores
	Generic health status	Euro-QOL5D (EQ-5D)	No significant relationship
		Utilisation of lumbar spine treatment (last 6 months), physiotherapy attendance, immunisation history, medication use, employment, days of work missed	Adequate HL group used more medications and consulted a specialist more frequently than limited HL group Limited HL group reported more individual visits to chiropractor and had lower use of NSAIDs

**Table 4:** HL measures used and the components of HL they cover

HL measure	Functional HL	Communicative HL	Critical HL
Short-form Test of Functional Health Literacy in Adults (S-TOFHLA)	YES	NO	NO
Health Literacy Measurement Scale (HeLMS)	YES	YES	YES
Low Back Pain Knowledge Questionnaire	YES	NO	NO
Newest Vital Sign (NVS)	YES × 3	NO	NO
Health Literacy Assessment (HLA)	YES	NO	NO
“How confident are you filling out medical forms by yourself?” screening question	YES	NO	NO
“Never heard of it/Do not know much about it” questions from the NHIS (National Health Interview Survey)	YES	NO	NO
HELP questionnaire (health education literacy of patients with chronic musculoskeletal diseases)	YES	YES	YES
3 screening questions from Brief Questions to Identify Patients with Inadequate Health Literacy	YES	NO	NO
Number of studies—HL component assessed (%)	11 (73.3%)	2 (13.3%)	2 (13.3%)

In their 2010 paper, Briggs et al. [10] used the Short-form Test of Functional Health Literacy in Adults (S-TOFHLA), which comprises of two prose passages and four items testing numeracy. It is a validated assessment of functional HL with good internal reliability—Cronbach’s alpha 0.68 for the 4 numeracy items [42] and 0.97 for the reading comprehension items [13, 42, 43]. Spearman’s correlation coefficient between the S-TOFHLA and the Rapid Estimate of Adult Literacy in Medicine (REALM) was 0.80 [42]. Briggs et al. [10] also used telephone interviews to assess HL by asking participants on how they sought, understood and utilised LBP information.

Subsequently in their 2011 paper, Briggs et al. used the Health Literacy Measurement Scale (HeLMS) [41], a psychometrically tested tool with good internal consistency (Cronbach’s alpha > 0.82), and validity (confirmatory factor analysis showing good fit for seven domains and moderate fit for one) [44]. It goes beyond functional HL to include communication skills, computation skills, and social support, thereby overcoming limitations of the S-TOFHLA [41, 45, 46]. The HeLMS sets out to assess “overall capacity to seek, understand and use health information within the healthcare setting” by asking questions such as “Are you able to see a doctor when you need to?” [41]. By doing so it attempts to assess all three domains of health literacy: functional, communicative, and critical.

The Newest Vital Sign (NVS) was the most frequently used HL measure (Table 3), utilised by Devraj, Herndon and Griffin, Al-Eisa, Buragadda and Melam, and Glassman et al. [12, 47, 48]. The NVS is convenient to use and has a sensitivity equivalent to the TOFHLA for identifying inadequate HL—with an area under the ROC curve (AUROC) of 0.88, using the TOFHLA as the gold standard [42]. It is reported to have a Cronbach’s alpha of 0.76 [42, 49], and is a widely used assessment of functional HL, with six questions regarding a standardised ice cream nutrition label [50].

Farin, Ullrich and Nagl developed the HELP questionnaire (Health Education Literacy of Patients with chronic musculoskeletal diseases), an 18-item assessment that aims to summarise a patient’s reported communication and comprehension difficulties in health education and treatment [11]. The questions were classified as “comprehension of medical information” (assessing functional HL), “communicative competence in provider interactions” (assessing communicative HL), and “applying medical information” (assessing critical HL). Questions such as “How much difficulty did you have communicating your own expectations and wishes in terms of your therapy?” were scored on Likert scales anchored from 1 to 5, with lower values indicating a higher HL. The resulting questionnaire’s psychometric properties were deemed to be good (Cronbach’s alpha 0.88 to 0.95, unidimensionality and Rasch model fit established) [11].

Camerini and Schulz [51] interpreted HL based on scores from the Low Back Pain Knowledge Questionnaire (LKQ). The Questionnaire involved multiple-choice questions on topics such as the aetiology and management of LBP. Although the LKQ did not set out to be a direct measure of HL, its focus was on declarative and procedural knowledge which Camerini and Schulz argued to be acquired using functional HL [51]. Hence the LKQ was used as a surrogate measure of functional HL. The LKQ was assessed with both intra-observer and inter-observer reproducibility (Spearman's correlation coefficient and intra-class correlation coefficient ranging from 0.61 to 0.95) and internal consistency (Cronbach’s alpha ranging from 0.71 to 0.77) [52].

Measurement of functional HL alone is also seen in other studies. Burke, Nahin and Stussman used the response option “Never heard of it/Do not know much about it” from the National Health Interview Survey, arguing that this serves as an indicator of health knowledge which in turn is a correlate of functional HL [53]. MacLeod et al. used a validated single-item screener “How confident are you filling out medical forms by yourself?” as a measure of functional HL [54]. This had an AUROC of 0.82 for detecting limited HL, and 0.79 for detecting limited or marginal HL, when referenced against the REALM functional HL measure [55].

Köppen et al. used HL questions taken from the Brief Questions to Identify Patients with Inadequate Health Literacy [20], a screening tool for functional HL validated against the S-TOFHLA [56]. These included the questions “how often do you have someone help you read hospital materials” (AUROC 0.87), “how confident are you filling out medical forms by yourself” (AUROC 0.80) and “how often do you have problems learning about your medical condition because of difficulty understanding written information?” (AUROC 0.76) [20, 56].

In addition to the NVS mentioned above, Glassman et al. also used The Health Literacy Assessment, a 10-item self-administered questionnaire using items selected from the computerized Health LiTT measure [47]. The Health Literacy Assessment (Health LiTT) is a validated tool for functional HL that reportedly meets or exceeds psychometric standards, with good reliability (Cronbach’s alpha 0.83–0.91) and good evidence for unidimensionality (correlation of 0.90–0.95 on confirmatory factor analysis) [57]. It assesses HL via three sections: Prose, Document and Quantitative [57]. The Prose section asks participants to fill in missing words in a cloze test passage, while the Document section consists of multiple-choice questions regarding images such as a prescription label. The Quantitative section also uses multiple-choice questions requiring arithmetic computation.

### Summary of LBP outcomes retrieved from included studies

HL was associated with a wide range of outcomes (Table 5). Five overarching categories summarising the studied LBP health outcomes were identified via framework method analysis [35, 36]:

**Table 5:** Health outcome measures used by category

Pain	Disability	Behaviour	Knowledge and beliefs	Resource utilisation
Health outcome measures employed (number of times)				
Numerical rating scale (3)	Oswestry Disability Index (4)	Coping Skills Questionnaire (2)	Back Pain Beliefs Questionnaire (2)	Utilisation of medications (3)
Visual Analogue scale (2)	Euro-QOL5D (1)	Fear Avoidance Beliefs Questionnaire (3)	Modified Consumer Assessment of Healthcare Providers and Systems survey (1)	Utilisation of healthcare appointments (9)
6 item Chronic Pain Grading Scale (1)	6 item Chronic Pain Grading Scale (1)	Psychological Empowerment Scale (1)	One-item measure—How would you rate your health? (1)	Healthcare cost—expenditure/workdays missed/affordability (6)
Short-form McGill Pain Questionnaire (1)		Modified Patients’ Perceived Involvement in Care Scale (1)		
Pain duration in months (1)				
Oswestry Disability Index (4)				
Euro-QOL5D (1)				
Others* (2) *LBP episodes in last 1 year	Others* (2) *Pain impact (intrusion on regular daily and recreational activities)	Others* (1) *Health Behaviours (Activity level, smoking status, alcohol consumption level, body mass index, flu immunisation in last 12 months, use of pneumonia vaccine)	Others* (3) *Telephone interviews (2), and 12-item survey developed by authors (1)

- Pain
- Disability
- Behaviour
- Knowledge and Beliefs
- Resource Utilisation

#### Pain

Seven studies involved data on pain [10, 12, 20, 41, 47, 48, 51], using eight different measures (Table 5). Pain intensity was the most frequently measured aspect, with three studies (Briggs et al., Briggs et al., Glassman et al.) using the Numerical Rating Scale and two (Devraj, Herndon and Griffin, Köppen et al.) using the Visual Analogue Scale (Table 5). Pain intensity was also quantified as a sub-component of the Chronic Pain Grading [51], the Short-form McGill Pain Questionnaire [20], the Oswestry Disability Index (ODI) [10, 41, 47, 48], and the Euro-QOL5D [47]. In addition, the Short-form McGill Pain Questionnaire assesses the nature of pain [58], while other studies looked at pain duration and frequency [10, 20].

#### Disability

Five studies involved data on disability [10, 41, 47, 48, 51]. Four studies (Briggs et al., Briggs et al., Glassman et al., Al-Eisa, Buragadda and Melam) used the ODI, a spinal disorder-specific measure of disability which quantifies the difficulty faced in areas such as personal care, movements (e.g. lifting, walking, sitting), and lifestyle (e.g. sex life, travel) [59]. Additionally, Glassman et al. [47] used the Euro-QOL5D (EQ-5D) which, in addition to mobility, self-care and activities of daily living, also screens for anxiety and depression. Both of Briggs et al.’s studies [10, 41] supplemented the ODI with an assessment of disability by asking participants on the amount of intrusion one faces in daily and recreational activities. Lastly, the Chronic Pain Grading Scale also asks about functionality using questions such as “In the past 3 months, how much has this pain interfered with your daily activities (e.g. getting dressed, doing shopping)” [51].

#### Behaviour

Five studies collected data on patient behaviours [10, 41, 48, 51, 53], involving five forms of health outcome measures (Table 5). The Fear Avoidance Beliefs Questionnaire was most commonly used [10, 41, 48], and asks participants how much they think areas of physical activity and work would affect their LBP [60]. Briggs et al. [10, 41] assessed pain catastrophizing with the Coping Skills Questionnaire. Camerini and Schulz [51] assessed patient empowerment and involvement with two scales, the Psychological Empowerment Scale and Modified Patients’ Perceived Involvement in Care Scale respectively. Burke, Nahin and Stussman [53] studied the association between HL and health behaviours such as physical activity level and smoking status.

#### Knowledge and beliefs

Four studies gathered data on patient knowledge and beliefs [15–17, 25], utilising five different health outcome measures. Briggs et al. [10, 41] used the Back Pain Beliefs Questionnaire, which consists of 14 questions exploring beliefs regarding issues such as the management and prognosis of back trouble [61]. They also conducted telephone interviews to understand participant’s beliefs regarding the aetiology and course of their LBP. MacLeod et al. [54] used the Modified Consumer Assessment of Healthcare Providers and Systems survey to assess patient dissatisfaction in areas such as general healthcare and doctors. Farin, Ullrich and Nagl [11] used a single-item measure—“How would you rate your health?” to evaluate participant beliefs on their health status. Finally, Devraj, Herndon and Griffin [12] developed a 12-item survey based on pre-existing pain guidelines and literature to assess the pain awareness and medication knowledge of their participants.

#### Resource utilisation

Four studies involved data on resource utilisation [47, 51, 53, 54]. A wide variety of resources were studied, and we broadly grouped these outcomes (Table 5) into utilisation of medications, utilisation of healthcare appointments (e.g. lumbar spine treatment, physiotherapy), and healthcare costs (e.g. expenditure, workdays missed). Of these, utilisation of healthcare appointments was measured the most—in nine occasions, while healthcare costs were measured six times, and utilisation of medications was measured thrice (Table 5).

### Comparison of included LBP health outcomes against the COS for clinical trials in non-specific LBP

A total of 50 health outcome measures were utilised across the ten studies reviewed. Of these, 14 (28%) were deemed to be directly related to those in the COS but were limited to two outcomes “pain intensity’ and “physical functioning” [22] (Table 6). The Pain Numerical Rating Scale, Pain Visual Analogue Scale, and Short-form McGill Pain Questionnaire directly addressed the core outcome of “pain intensity”, while the ODI, Euro-QOL5D, Chronic Pain Grading Scale, and questions on intrusion of daily and recreational activities [10, 41] directly addressed the outcome “physical functioning”. Measures on pain duration and frequency were only indirectly related to the COS. The COS outcome “health-related quality of life” had the greatest number of measures indirectly addressing it (Table 6). This was because three of the five overarching categories of health outcomes (behaviour, knowledge and beliefs, and resource utilisation) were found to be assessments of the “impact on physical, psychological and social domains of health”—i.e. the COS’ definition of “health-related quality of life” [22]. The COS outcome “Number of Deaths” was not explored in any of the included studies.

**Table 6:** Summary of number of health outcome measures directly and indirectly related to the COS for clinical trials in non-specific low back pain

COS	Directly related outcomes	Indirectly related outcomes
Pain intensity	6	3
Physical functioning	8	0
Health-related quality of life	0	33
Number of deaths	0	0
Totals (%)	14 (28%)	36 (72%)

### Association between HL and LBP health outcomes

Although not a primary aim of this scoping review, we briefly detail here findings on the association between HL and LBP health outcomes as a snapshot of existing literature (Table 3). Out of six studies analysing the relation between HL and levels of pain and disability [10, 12, 20, 41, 47, 48], only two found a significant association, particularly in the area of pain intensity [20, 47]. On behavioural impact, HL had no significant associations with fear avoidance [10, 41], pain catastrophising [10, 41], and psychological empowerment [51]. However, patients with low HL scores were found to have a less active lifestyle [53]. Considering patient knowledge and beliefs, those with lower HL scores had more difficulty identifying types and sources of treatment for LBP [12] and were more dissatisfied with their care [54]. However, no significant association was found between HL and beliefs about one’s future with LBP [10, 41]. Regarding resource utilisation, it appears that low HL scores were associated with higher utilisation of curative or symptomatic treatment (e.g. emergency room visits), and lower utilisation of preventive medicine (e.g. flu vaccinations) [54].

## Discussion

We will now discuss our results in the context of the three objectives and the implications for evidence and future research i.e. (1) The health literacy measures currently employed for low back pain and the aspects of health literacy they include; (2) The low back pain health outcomes included in such work; (3) The extent to which these health outcomes reflect the Core Outcome Set for Clinical Trials in Non-Specific Low Back Pain.

The scoping review yielded ten relevant studies. Among the nine different measures of HL used, all involved the study of functional HL. The 50 measures of LBP health outcomes could be grouped into five thematic categories, namely: Pain, Disability, Behaviour, Knowledge and Beliefs, and Resource Utilisation. However, most of these health outcomes did not seek to directly satisfy the COS for Clinical Trials in Non-Specific LBP.

### Health literacy measures employed

The studies included in this scoping review adopted a wide variety of measures to document HL (nine measures used) and health outcomes (50 measures used). This hampered the comparison of results across studies and the development of a comprehensive evidence-base despite the development of the COS [22].

Despite expanding the search and using a more open search criteria, this study only included seven studies in addition to those in the systematic review by Edward et al. [27]. The dearth of relevant studies in this scoping review highlights the continuing lack of evidence of the relationship between HL and LBP health outcomes.

Although no restrictions were placed on the year of publication, all relevant studies were published in 2010 or later, suggesting that interest in the association between HL and LBP is relatively recent. This may reflect HL being a relatively new concept within healthcare [62], and the growing interest in LBP as it contributes to rapidly rising healthcare expenditure [2, 63]. For instance, from 1996 through 2013, US expenditure on low back and neck pain rose by an estimated USD 57.2 billion, becoming the third-highest healthcare spending on a single condition in 2013 [63].

Despite no language or country restrictions being placed on the search, all studies were conducted in high income countries—as defined by the 2021 World Bank classification of economies [64]. This may be a barometer of societal readiness to integrate HL into LBP management. Most pressingly, there is a notable absence of research attempting to draw associations between LBP and HL in LMICs and collectively in Asia, Africa, and South America. This is in keeping with previous epidemiological studies remarking that LBP monitoring and research is largely restricted to high income countries, while being under-researched in LMICs [23, 24]. Alongside this is an increasing recognition of the need to develop and use culturally sensitive HL tools [65].

### Outcome measures used

Few studies incorporated the four outcome domains of the COS (pain intensity, physical functioning, health related quality of life, and number of deaths); only 14 of the 50 health outcome measures used did so. Moreover, these 14 measures were limited to the two core outcome domains of pain intensity and physical functioning (Table 5). This suggests a divergence of opinions on what is deemed as a key health outcome for people with LBP. This is concerning given that development of the COS incorporated a comprehensive range of views via a Delphi process with patients, care providers and researchers, a review by panellists who had published extensively on LBP, and by a four-continent International Steering Committee [22].

Given the methodology in developing the COS, future studies on LBP are strongly recommended to adopt them. The benefit of adopting the COS is twofold. Firstly, it allows future studies on LBP to have a more robust foundation to build upon. Secondly, the use of common health outcomes allows secondary research to have more compatible data for the comparison of findings. Overall, this allows for the development of a more rigorous evidence base. Also of note, the authors of the COS have subsequently argued for the inclusion of the 24-item Roland-Morris Disability Questionnaire for measuring physical functioning, and the Short Form Health Survey 12 and 10-item PROMIS Global Health form for measuring health-related quality of life [66]. However, none of these tools were used in the included studies (Table 2).

### Implications for future research

As highlighted in our findings, several limitations were noted in the literature with implications for future research design, specifically regarding study design, measures used and included study populations. It is of utmost importance that future research takes these findings into account in curbing the limitations of future research.

By solely employing cross-sectional study designs, the longitudinal relationship between HL and LBP outcomes was not explored. There was also a lack of evidence regarding the efficacy and implementation of HL interventions for people with LBP. Although a mixed-methods approach is preferable to holistically evaluate the complex construct of HL [67], only the 2010 study by Briggs et al. utilised quantitative and qualitative approaches [10].

Another limitation of in terms of study design was that the primary studies relied heavily on patient reported outcome measures (PROMs), which may be biased by one’s physical and psychological states, along with one’s memory, willingness, and ability to answer the questions. This may influence one’s ability to give accurate self-assessments of health status [68]. This limitation could be overcome by the concurrent use of objective markers (e.g. functional tests), diagnostic imaging (e.g. functional magnetic resonance imaging), and/or observer reported outcomes [69, 70].

Many studies also had limitations in terms of the HL measure used. Communicative and critical HL measures were under-investigated. HeLMS, and the questionnaires used by Camerini and Schulz [51], MacLeod et al. [54] and Burke, Nahin and Stussman [53] have not been used as widely as the S-TOFHLA and NVS, and their content validity in other settings requires confirmation. We were also unable to find psychometric data for the HL measure used by Burke, Nahin and Stussman [53]. It is recommended that future works reinforce their HL data by employing the use of HL measures with good psychometric validity and reliability. While a varied questioning style is likely to result in a more complete assessment of HL, measures tended to focus purely on either objective response (e.g. S-TOHHLA and NVS) or subjective replies (e.g. HeLMS). Ideally future studies on HL should use assessment tools that cover all three domains of HL as well as have vigorous validation in the setting employed.

Study population characteristics were also a source of limitation in the studied literature. Briggs et al. [10, 41] faced a limited distribution of HL, hampering their efforts to analyse the presence of associations between health literacy scores and other outcomes. Studies which excluded patients based on language literacy potentially excluded lower HL participants. If basic language proficiency is required to obtain self-reported patient outcomes, this may come at the cost of excluding certain sectors of the population. The use of translators or pictorial questionnaires need to be explored to enable the inclusion of participants who may be experiencing vulnerability, for example those facing communication barriers or multimorbidity [71, 72].

Responder bias through self-selection was another common limitation in terms of study population design. This is important in the context of HL studies, as low HL patients with lesser ability to communicate well with their healthcare provider may have a tendency to decline study involvement [73]. This limitation may potentially be mitigated using retrospective and anonymised data, rather than depend on the voluntary actions of patients.

### Strengths and limitations

The strengths of this review include the wide search strategy, involving 13 bibliographic databases with no search limiters or filters. By including studies on all forms of LBP health outcomes associated with HL, this review was able to build on the work of Edward et al. [27]. This review also followed best practices in the Joanna Briggs Institute methodology for conducting a scoping review, and the PCC mnemonic was adopted [30]. Expert opinion in LBP, HL, scoping reviews, and literature searching was also consulted. This was in line with best practice recommendations by the Institute of Medicine (US) Committee on Standards for Systematic Reviews of Comparative Effectiveness Research [74], as well as Arksey and O’Malley’s and Levac et al.’s frameworks for scoping reviews [29, 34].

Nevertheless, the search strategy was not without its flaws. The use of MeSH terms was done with the aim of improving reproducibility of results, especially with searches repeated periodically in this study. However, this ran the risk of missing out on recent articles not yet indexed. Furthermore, a more broadly defined strategy using additional synonyms for MeSH terms could have broaden the search even more. Grey literature was also excluded, which given the paucity of evidence in the field, could well have enriched this study’s findings [75].

The studies by Devraj, Herndon and Griffin [12], MacLeod et al. [54], Farin, Ullrich and Nagl [11], and Köppen et al. [20] did not have a solely LBP population, but were included as the LBP population made up at least 10% of the overall study. This was pre-determined as the cut-off percentage for eligibility into this review. This cut off has been used as a marker for significance in other studies [76, 77], but caution may be needed when interpreting the results of these studies.

The heterogeneity of measures employed, as well as the paucity of relevant studies, made it difficult to compare findings across studies and provide firm conclusions on the association between HL and each LBP health outcome. Thus, we were unable to draw strong evidence-based conclusions on this. We also note that classifying measurement tools into functional, communicative and critical HL as proposed by Nutbeam [37] is an imperfect method of HL classification, given the wide range of HL definitions employed and the fact that such a classification may not be the intention of the various measures. However, the benefit of using the classification in this review is that it has an ascending level of “difficulty”, thus capturing a sense of the complexity and dimensions of HL each measurement tool was seeking to assess, whether implicitly or explicitly.

The protocol was not registered a priori, leading to potential bias. However, as stated, no changes to the protocol were necessitated during the review process and data extraction remained per protocol. Piloting of the data extraction form was also not included. However, these are not requirements of a scoping review and were deemed unnecessary to fulfil the study objectives.

In terms of stakeholder involvement, while experts (clinicians and researchers) in the field of HL and LBP are members of the research team, patients were not consulted. There is growing evidence of the value of patient and public involvement at all stages of the research process [78], and the importance of how best to operationalise this within diverse cultural contexts [79, 80]. While deemed to be non-essential at this scoping stage, involving patients in the development of research questions would be essential to further work into the impact of HL on LBP health outcomes.

## Conclusions

The ten relevant studies included in this review yielded a total of nine different measures of HL and 50 measures of LBP health outcomes. Most health outcomes evaluated by the included studies did not seek to directly satisfy the Core Outcome Set for Clinical Trials in Non-Specific LBP. The wide variety of measures used hampers efforts to form conclusive relationships between HL and LBP outcomes, and precludes the use of a meta-analysis approach. To allow for comparison across findings and the development of a rigorous evidence base, future work should seek to address the Core Outcome Set for Clinical Trials in Non-Specific LBP. Furthermore, research thus far has focused on a narrow range of populations and there is an urgent need to broaden the evidence-base to include those where LBP morbidity is high, but data is lacking. As noted above, this is especially so in LMICs. Such work demands the incorporation of comprehensive measures of health literacy that have both generic and culturally sensitive components.

## Data Availability

The datasets used and/or analysed during the current study are available from the corresponding author on reasonable request.

## Appendix 1: Search strategy overviews

**Table:**

Database: Pubmed 1946 to Present. (health literacy[MeSH Terms]) AND ((((((((((((((((((Back Pain[MeSH Terms]) OR Back Ache[MeSH Terms]) OR Back Pain with Radiation[MeSH Terms]) OR Back Pain without Radiation[MeSH Terms]) OR Backache[MeSH Terms]) OR Vertebrogenic Pain Syndrome[MeSH Terms]) OR Low Back Pain[MeSH Terms]) OR Low Back Ache[MeSH Terms]) OR Low Back Pain, Mechanical[MeSH Terms]) OR Low Back Pain, Posterior Compartment[MeSH Terms]) OR Low Back Pain, Postural[MeSH Terms]) OR Low Back Pain, Recurrent[MeSH Terms]) OR Low Backache[MeSH Terms]) OR Lower Back Pain[MeSH Terms]) OR Lumbago[MeSH Terms]) OR Mechanical Low Back Pain[MeSH Terms]) OR Postural Low Back Pain[MeSH Terms]) OR Recurrent Low Back Pain[MeSH Terms]) No search filters used (e.g. specifying years, language). Database: Google Scholar “Health Literacy” AND (“Back Pain” OR “Back Ache” OR “Back Pain with Radiation” OR “Back Pain without Radiation” OR “Vertebrogenic Pain Syndrome” OR “Low Back Pain” OR “Low Back Ache” OR “Mechanical Low Back Pain” OR “Low Back Pain Posterior Compartment” OR “Postural Low Back Pain” OR “Recurrent Low Back Pain” OR “Low Backache” OR “Lower Back Pain” OR “Lumbago” OR “Mechanical Low Back Pain” OR “Postural Low Back Pain” OR “Recurrent Low Back Pain”) No search filters used (e.g. specifying years, language). Analysed from page 1 to 520 of the above Google Scholar query.	

## Appendix 2: PRISMA-ScR Checklist (Tricco et al. [32])

**Table:**

Section	Item	PRISMA-ScR checklist item	Reported on page #
Title			
Title	1	Identify the report as a scoping review	1
Abstract			
Structured summary	2	Provide a structured summary including, as applicable: background, objectives, eligibility criteria, sources of evidence, charting methods, results and conclusions that relate to the review question(s) and objective(s)	2–3
Introduction			
Rationale	3	Describe the rationale for the review in the context of what is already known. Explain why the review question(s)/objective(s) lend themselves to a scoping review approach	4–6
Objectives	4	Provide an explicit statement of the question(s) and objective(s) being addressed with reference to their key elements (e.g., population or participants, concepts and context), or other relevant key elements used to conceptualize the review question(s) and/or objective(s))	6
Methods			
Protocol and registration	5	Indicate if a review protocol exists, if and where it can be accessed (e.g., web address), and, if available, provide registration information including registration number	7–10
Eligibility criteria	6	Specify the characteristics of the sources of evidence (e.g., years considered, language, publication status) used as criteria for eligibility, and provide a rationale	8
Information sources	7	Describe all information sources (e.g., databases with dates of coverage, contact with authors to identify additional sources) in the search, as well as the date the most recent search was executed	7
Search	8	Present the full electronic search strategy for at least one database, including any limits used, such that it could be repeated	7
Selection of sources of evidence	9	State the process for selecting sources of evidence (i.e., screening, eligibility) included in the scoping review	8–9
Data charting process	10	Describe the methods of charting data from the included sources of evidence (e.g., piloted forms; forms that have been tested by the team before their use, whether data charting was done independently, in duplicate) and any processes for obtaining and confirming data from investigators	8–9
Data items	11	List and define all variables for which data were sought and any assumptions and simplifications made	8–9
Critical appraisal of individual sources of evidence	12	***If done***, provide a rationale for conducting a critical appraisal of included sources of evidence; describe the methods used and how this information was used in any data synthesis (if appropriate)	NA
Summary measures	13	Not applicable for scoping reviews	NA
Synthesis of results	14	Describe the methods of handling and summarizing the data that were charted	9
Risk of bias across studies	15	Not applicable for scoping reviews	NA
Additional analyses	16	Not applicable for scoping reviews	NA
Results			
Selection of sources of evidence	17	Give numbers of sources of evidence screened, assessed for eligibility, and included in the review, with reasons for exclusions at each stage, ideally using a flow diagram	10
Characteristics of sources of evidence	18	For each source of evidence, present characteristics for which data were charted and provide the citations	10
Critical appraisal within sources of evidence	19	***If done***, present data on critical appraisal of included sources of evidence (see item 12)	NA
Results of individual sources of evidence	20	For each included source of evidence, present the relevant data that were charted that relate to the review question(s) and objective(s)	11, 14, 17
Synthesis of results	21	Summarize and/or present the charting results as they relate to the review question(s) and objective(s)	10–18
Risk of bias across studies	22	Not applicable for scoping reviews	NA
Additional analyses	23	Not applicable for scoping reviews	NA
Discussion			
Summary of evidence	24	Summarize the main results (including an overview of concepts, themes, and types of evidence available), explain how they relate to the review question(s) and objectives, and consider the relevance to key groups	19–23
Limitations	25	Discuss the limitations of the scoping review process	23–25
Conclusions	26	Provide a general interpretation of the results with respect to the review question(s) and objective(s), as well as potential implications and/or next steps	25–26
Funding			
Funding	27	Describe sources of funding for the included sources of evidence, as well as sources of funding for the scoping review. Describe the role of the funders of the scoping review	27

## Appendix 3: Key excluded sources with rationale for their exclusion

**Table:**

References	Rationale for exclusion
Slater et al. [81]	Study did not attempt to draw a relationship between HL and LBP health outcomes
Larsen et al. [82]	Lack of significant LBP population
Khoshnevisan et al. [83]	Lack of explicit study of HL
Kim [84]	Lack of significant LBP population
Roth et al. [85]	Lack of explicit study of HL Lack of significant LBP population
Hardie et al. [86]	Lack of significant LBP population
Schulz et al. [87]	Lack of explicit study of HL
Rabenbauer and Mevenkamp [88]	Study did not attempt to draw a relationship between HL and LBP health outcomes

## Abbreviations

HL: Health literacy
LBP: Low back pain
COS: Core Outcome Set
S-TOFHLA: Short-form Test of Functional Health Literacy in Adults
HeLMS: Health Literacy Measurement Scale
NVS: Newest Vital Sign
FABQ: Fear Avoidance Beliefs Questionnaire
